# Community knowledge, attitude, and practice, incidence of suspected cases, and epidemiological distribution of rabies in humans and animals in Southwest Shewa zone, Oromia, Ethiopia

**DOI:** 10.3389/fvets.2025.1448448

**Published:** 2025-04-08

**Authors:** Tegegn Dilbato Dinbiso, Abebe Tibebu Mekonnen, Tolesa Negasa Olana, Gudina Mekonnen Ayana, Kebede Abdisa Kelbesa, Hana Dejene Deso, Segni Bedasa Gudina, Moges Kidane Biru, Mulatu Ayana Hordofa, Mekonnen Sorsa Berecha

**Affiliations:** ^1^Department of Veterinary Science, School of Veterinary Medicine, Ambo University, Guder Mamo Mezemir Campus, Ambo, Ethiopia; ^2^Department of Livestock and Fisheries Development Core Work Process, Ilu Gelan District Agriculture Office, Ejaji, Oromia, Ethiopia; ^3^Department of Land Administration and Surveying, School of Natural Resource Management, Ambo University, Guder Mamo Mezemir Campus, Ambo, Ethiopia; ^4^Department of Soil, Water, and Ecosystem Sciences, Global Food Systems Institute, University of Florida, Gainesville, FL, United States; ^5^Department of Public Health, College of Medicine and Health Science, Ambo University, Ambo, Ethiopia; ^6^Department of Veterinary Laboratory Technology, School of Veterinary Medicine, Ambo University, Guder Mamo Mezemir Campus, Ambo, Ethiopia

**Keywords:** animal, epidemiological distribution and knowledge, human, attitude and practice, incidence, rabies

## Abstract

**Background:**

Rabies remains a major public and veterinary health problem in most developing countries, including Ethiopia. Despite its importance in public health, no systematic study has been conducted in the area. Therefore, this study aimed to assess the knowledge, attitude, and practice and to estimate the incidence and spatial distribution of rabies in humans and animals in the Southwest Shewa zone of Oromia, Ethiopia.

**Methods:**

A questionnaire-based cross-sectional and retrospective study designs were employed among 422 randomly selected informants potentially at risk of rabies. Data were collected using a semi-structured questionnaire. The study utilized retrospective data from individuals admitted for rabies between 2017 and 2021 to analyze and map the epidemiological distribution of the disease using ArcGIS. Furthermore, descriptive statistics and logistic regression models were employed to analyze the data.

**Results:**

This study revealed that the level of good knowledge, positive attitude, and good prevention practices toward rabies among the communities was 58.3, 47.9, and 54.2%, respectively. The proportion of respondents who own dogs (OR = 1.7, 95% CI: 1.050–2.873, *p* = 0.032), live in urban areas (OR = 10.7, 95% CI: 1.106–103, *p* = 0.042), and have a higher degree of education (OR = 2.4, 95% CI: 1.061–5.513, *p* = 0.036) were statistically significantly associated with good knowledge scores toward rabies. Private workers and urban residents were statistically associated with positive attitude scores on rabies (*p* < 0.05). Living in urban areas (OR = 2.9, 95% CI: 1.596–5.407, *p* = 0.000) and Weliso district (OR = 10, 95% CI: 4.099–24.560, *p* = 0.000) had good prevention practice scores toward rabies. A total of 529 suspected rabies cases with an overall incidence of 44.9 cases per 100,000 population in humans and 127 suspected rabies cases with an overall incidence of 3.4 cases per 100,000 population in animals were registered from 2017 to 2021.

**Conclusion:**

The level of knowledge, positive attitude, and good practice toward rabies was found to be inadequate. Therefore, awareness creation programs and effective and well-organized prevention and control measures should be employed, with a special focus on identified risk factors, to reduce disease burdens through a One Health (OH) approach.

## 1 Introduction

Zoonosis is an infectious disease that can be spread from vertebrate animals to humans or vice versa. Several routes can transmit the disease: direct contact, through intermediate vectors such as ticks and mosquitoes, or food and water (1). The diseases account for approximately 75% of emerging infectious diseases and can be devastating to both human and animal health globally (2). A subset of those infectious diseases is referred to as “neglected zoonotic disease—NZDs” (3). Ethiopia is vulnerable to these diseases because a segment of the population lives near animals frequently in unsanitary conditions with limited access to healthcare (4, 5). Rabies is among the eight illnesses designated as NZDs by the World Health Organization (5). Rabies is a life-threatening zoonotic disease caused by a *Lyssavirus* in the family *Rhabdoviridae* with a worldwide occurrence and is transmitted mainly by carnivores to humans and livestock (6, 7). Although dog bites account for most human rabies cases globally, bats play an important role in the cycle of rabies transmission (8).

Today, rabies remains a major public and veterinary health problem in most developing countries, particularly in the tropical and subtropical regions of Africa and Asia (9). Rabies is endemic in Ethiopia, which kills 10,000 people each year (7). The challenges associated with rabies prevention in animals and humans remain unresolved. The contribution of attitudes and behavior of the community toward the prevention of rabies is poorly understood (9). There were more rabies-suspected cases (6/100,000) and human deaths (5/100,000), according to retrospective research carried out in several regions of Ethiopia (10–14); animal-related bites have also been conducted in Ethiopia as a whole (11). There are few studies attempted elsewhere to determine the level of awareness of rabies (12). Another survey by Aga et al. (13) pointed out a knowledge gap (56.15%), especially regarding rabies treatment, vaccination, and prevention in humans and animals.

Through collaborative, multisectoral efforts involving the human, veterinary, and environmental sectors, the Ethiopian government developed a nationwide rabies control and elimination strategy by 2018–2030. Mass dog vaccination is the main goal of the plan. Furthermore, the management of dog populations, prevention and control of rabies in wildlife, strengthening surveillance, and diagnostic capacities, information exchange between sectors, and human prevention through awareness and post-exposure prophylaxis are essential components of a comprehensive rabies prevention and control strategy (14). This scenario is related to the difficulties in fully eliminating rabies because of the disease’s widespread distribution, wide host range, high number of stray dogs, lack of access to treatment, and low levels of public and community awareness. According to Hagos et al. (15) and Laorujisawat et al. (16), studies on knowledge, attitude, and practice (KAP) in public health have been extensively used because of the principle that enhancing knowledge will lead to changes in attitudes and practices that lower the burden of disease. Furthermore, KAP research can be used to create public health awareness campaigns that provide baseline data for the development, implementation, and evaluation of national disease control programs (16).

Apart from repeated disease outbreaks, there is a lack of adequate data on rabies in humans and animals, and there was little research done on the disease’s dynamics and risk factors (17, 18). Due to insufficient surveillance and a lack of trustworthy data, the burden and distribution data are incomplete and not updated frequently (19). Furthermore, using a geographic information system (GIS) to map the epidemiological distribution of zoonotic rabies in space and time is critical in this setting for better allocation of resources for their prevention (20). Therefore, the present study was initiated to assess communities’ knowledge, attitude, and practice and map the epidemiological distribution in humans and animals in selected districts of the Southwest Shewa zone, Oromia, Ethiopia. Moreover, it aimed to determine the incidence of suspected cases from retrospective data (2017–2021) obtained from health centers and veterinary clinics.

## 2 Materials and methods

### 2.1 Study sites

**Figure 1:**
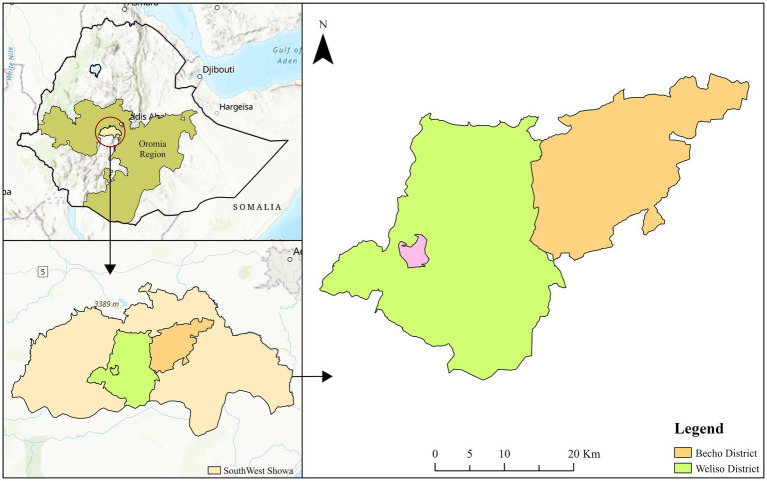
Map of the study area. Source: designed by (ArcGIS 10.4).

The study was conducted in the Weliso and Bacho districts of the Southwest Shewa zone, Oromia Regional State, Ethiopia. Weliso is the capital town of the zone, which is approximately 114 km southwest of Addis Ababa. The zone is located on Latitude 8° 36′ 33″ North and Longitude 38° 14′ 7.2″ East with an elevation of 2,227 meters above sea level. The annual rainfall ranges between 1,350 and 1,600 mm with annual temperatures of 15°C and 24°C, respectively. The zone is characterized by four typical agro-climatic seasons, namely, summer, which is the main rainy season, autumn, winter, and spring. The zone has a total human population of 1,101,129, of whom 556,194 are men and 544,935 are women; 149,878 or 13.61% of the population are urban inhabitants. A total of 233,916 households were counted in this zone, which resulted in an average of 4.71 persons per household and 227,102 housing units (21). The total livestock population of the zone is 1,308,606 cattle, 401,569 sheep, 317,287 goats, 65,363 horses, 30,965 mules, and 133,065 donkeys, which results in an average of 5.59 cattle per household, 1.71 sheep per household, 1.35 goats per household, 0.28 horses per household, 0.13 mules per household, and 0.57 donkeys per household. In addition, households keep other animals, such as dogs and cats, which are significant in the context of rabies transmission (23,927 dogs and 12,511 cats) (Southwest Shewa Zone Agricultural Office, 2021). The map of study districts is depicted in Figure 1.

### 2.2 Study population

The study populations were communities and animals (on rabies) found in two purposively selected districts of the Southwest Shewa zone, namely, Weliso and Bacho. As study participants, individuals admitted for rabies cases from 2017 to 2021 were included to map the epidemiological distribution of the disease. For the assessment of knowledge, attitude, and practice in the community, individuals from those four purposively selected peasant associations (PAs) of the districts were targeted.

### 2.3 Study design

A combination of cross-sectional and retrospective study designs was employed to investigate rabies in the study area. The cross-sectional questionnaire-based survey assessed the community’s knowledge, attitudes, and practices (KAP) regarding rabies. Meanwhile, the retrospective study design focused on mapping the epidemiological distribution of rabies cases by utilizing data from suspected individuals and patients who received post-exposure prophylaxis (PEP) for rabies. These records were extracted from registration books of hospitals, health centers, and veterinary clinics within the study area, covering the period from 2017 to 2021.

### 2.4 Sampling method and sample size determination

**Figure 2:**
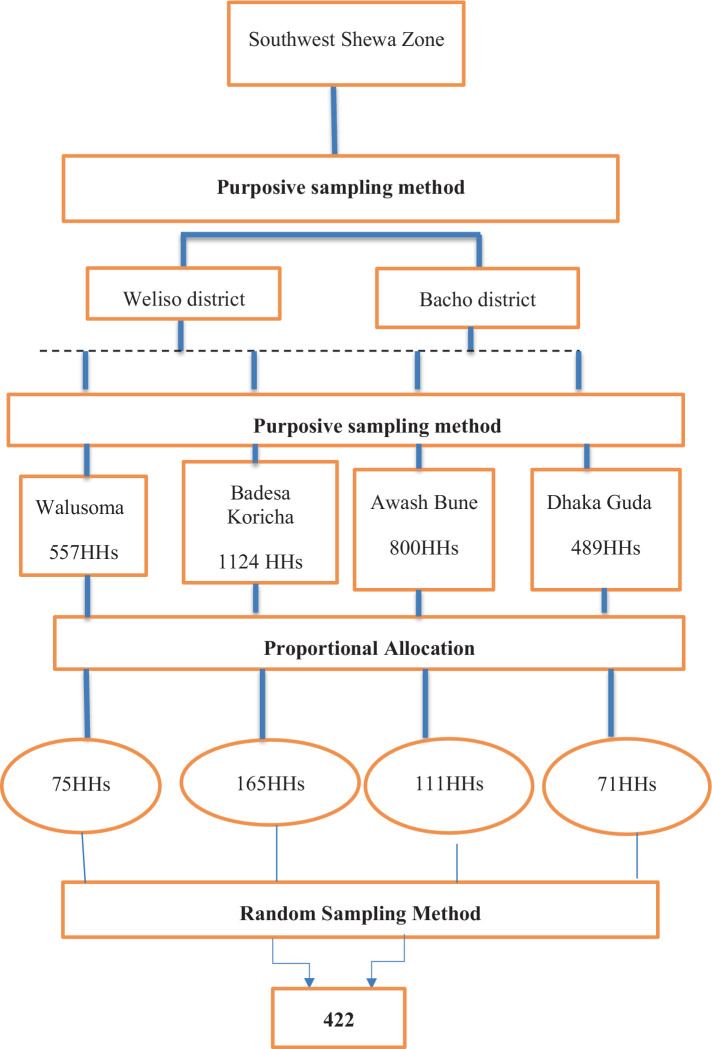
Conceptual framework for sampling method. Source: Own survey data (2021).

The study districts and peasant associations (PAs) (Badesa Koricha and Walusoma) from Weliso district and (Awash Bune and Dhaka Guda) from Bacho district were purposively selected based on the previous known burden of suspected rabies exposures. Finally, a random sampling technique was employed to select the study unit. Therefore, 422 respondents were selected from a total of 2,970 households (HHs), as presented in Figure 2.

The required sample size for this study was estimated by considering the study reporting proportion/percentage of peoples’ knowledge level 46.1%, attitude level 56.5%, and practice level 63.5% at Ambo town (22) with a 5% margin of error and 95% confidence level. Thus, the calculated sample size for knowledge, attitude, and practice will be 382, 384, and 356, respectively. The larger sample size among the knowledge, attitude, and practice is taken as appropriate, which is 384. Therefore, the total sample size to select respondents was computed by adding a 10% non-response rate; thus, the total sample size included 422 respondents, as indicated by using the Trusfield formula (23).


$$n=\frac{1.96^{2}\mathrm{Pexp}\;\left(1-\mathrm{Pexp}\right)}{d^{2}}$$

where *n* = required sample size, Pexp = expected prevalence, and *d*^2^ = desired absolute precision.

### 2.5 Data collection

The questionnaire was prepared from previous literature (9, 13, 15, 16, 19, 22) on the subject matter and analyzed by other individuals for quality. A pre-tested, semi-structured questionnaire was provided to assess knowledge, attitude, and practice in the communities. The questionnaire was developed in English and translated into the local language (Afaan Oromo). The questionnaire asked respondents for socio-demographic data such as age (18–30 years, 31–50 years, and 51–70 years), gender (male and female), residence (peri-urban, urban, and rural), religion (Christian and Muslim), level of education (Illiterate, 1–8 grade, 9–12 grade, and above 12 grade), occupation (government employee, private worker, and farmer), and previous exposure in their family (yes and no) (22).

Questions regarding the cause, source of information, fatal nature, clinical signs, reservoir hosts, commonly affected species of animals, and seasonal variations were asked for knowledge assessment. Concerning attitude, specific questions were prepared for the community on zoonotic nature, the timing of medical evaluation, prevention by vaccination, and drug preference for diseases. Practice mechanisms for the infected and dead animals from the diseases, dog vaccination, and dog management were asked of the participants to assess the practice of the community.

Retrospective data were collected from hospitals, health centers, and veterinary clinics from registered casebooks of admitted individuals for rabies cases, if any, from 2017 to 2021 consecutive years. The questionnaire was prepared to collect data from health centers and veterinary clinics. In humans, age, sex, residence, species bitten, owner of the animal, type of exposure, site of the bite, date of bite, date of report, season, and type of post-exposure treatment given were considered, while seasons of an outbreak, species of infected animals, season, sex, and source of the diseases were recorded in animals.

Furthermore, geographical coordinate systems of health centers (latitude, longitude, and altitude) were obtained using GPS from the study area to map the distribution of disease. By having the number of suspected rabies cases collected from the health centers of the district, the incidence of those diseases was calculated, and then, the map was generated depending on the level of incidence by using ArcGIS. The various functionalities of ArcGIS were used to map the zonal-level distribution of the selected rabies as per the procedures and protocol described by a previous study (18). To compute a zonal-level distribution of rabies, a database at district health centers was created using data obtained from the zonal health bureau. For accuracy and convenience purposes, the “Ethio-region” and “Southwest Shewa” shape files were obtained from the Africa Geo-portal website (23).

### 2.6 Operational definitions

*Good knowledge:* Respondents who scored more than 50% for the knowledge questions were referred to as having good knowledge, otherwise not.

*Positive attitude:* Respondents who scored more than 50% for the attitude questions toward rabies were referred to as having a positive attitude, otherwise not.

*Good practice:* Respondents who scored more than 50% for the practice questions toward rabies were referred to as having good practice, otherwise not.

*Human suspected exposure:* A person presenting for healthcare with a history of a bite, scratch, or contact with infectious material from a suspected, probable, or confirmed rabid animal.

*Animal suspected rabies case:* An animal that presents with any of the following signs: hypersalivation, paralysis, lethargy, unprovoked abnormal aggression (biting two or more people or animals and/or inanimate objects), abnormal vocalization, and diurnal activity of nocturnal species.

### 2.7 Data management and analysis

The data collected were entered and coded in a Microsoft Excel 2010 spreadsheet computer program. Descriptive statistics (percentage and frequency distribution) were employed to summarize the data using the Software Program for Social Science (SPSS) version 20. In addition, logistic regression was used to assess the associations between risk factors (dog ownership, previous exposure, source of information, age, sex, education, religion, residence, occupation, and districts) and KAP scores. Knowledge, attitude, and practice (KAP) scores were categorical variables generated from specific questions applied to measure them. In all cases, a 95% CI was employed to estimate sample results for the target population in the study area. The variables having a *p*-value of <0.25 on univariable logistic regression were transferred to multivariable logistic regression. *p*-values of less than 5% were considered statistically significant associations.

The KAP scoring method was implemented following the approaches outlined by Mapatse et al. (9), Sambo et al. (24), Ebuy et al. (25), and Abdulsalam et al. (26). Respondents’ knowledge of rabies was scored and graded on the 11-point scale. One point was awarded for a correct response, while a wrong response or I do not know response received no points. This gives a minimum score of “0” and a maximum score of “11” points. Those who scored ≥6 of 11 points were considered as having “good” knowledge, while those who scored <6 of 11 points were graded as having “poor” knowledge. Respondents’ attitude toward rabies was scored and graded on a 7-point scale. One point was awarded for a correct response, while a wrong response or I do not know response received no points. This gives a minimum score of “0″ and a maximum score of “7” points. Those who scored ≥4 of 7 points were considered as having a “positive” attitude, while those who scored <4 of 7 points were graded as having a “negative” attitude. Moreover, respondents’ practice on rabies was scored and graded on a 9-point scale. One point was awarded for a correct response, while a wrong response or I do not know response received no points. This gives a minimum score of “0” and a maximum score of “9” points. Those who scored ≥5 of 9 points were considered as having “good” practice, while those who scored <5 of 9 points were graded as having “poor” practice.

Geographical coordinate data were also included in the Excel file and then exported into the ArcGIS software. The necessary layers were added as years and areas. Finally, the map for the district was drawn up by pointing out the incidence of 5 years in the study area (27). The map of the incidence rate in each district was obtained by dividing the number of rabies patients by the total number of populations at risk in the districts.

## 3 Results

### 3.1 Socio-demographic profiles of study participants

**Figure 3:**
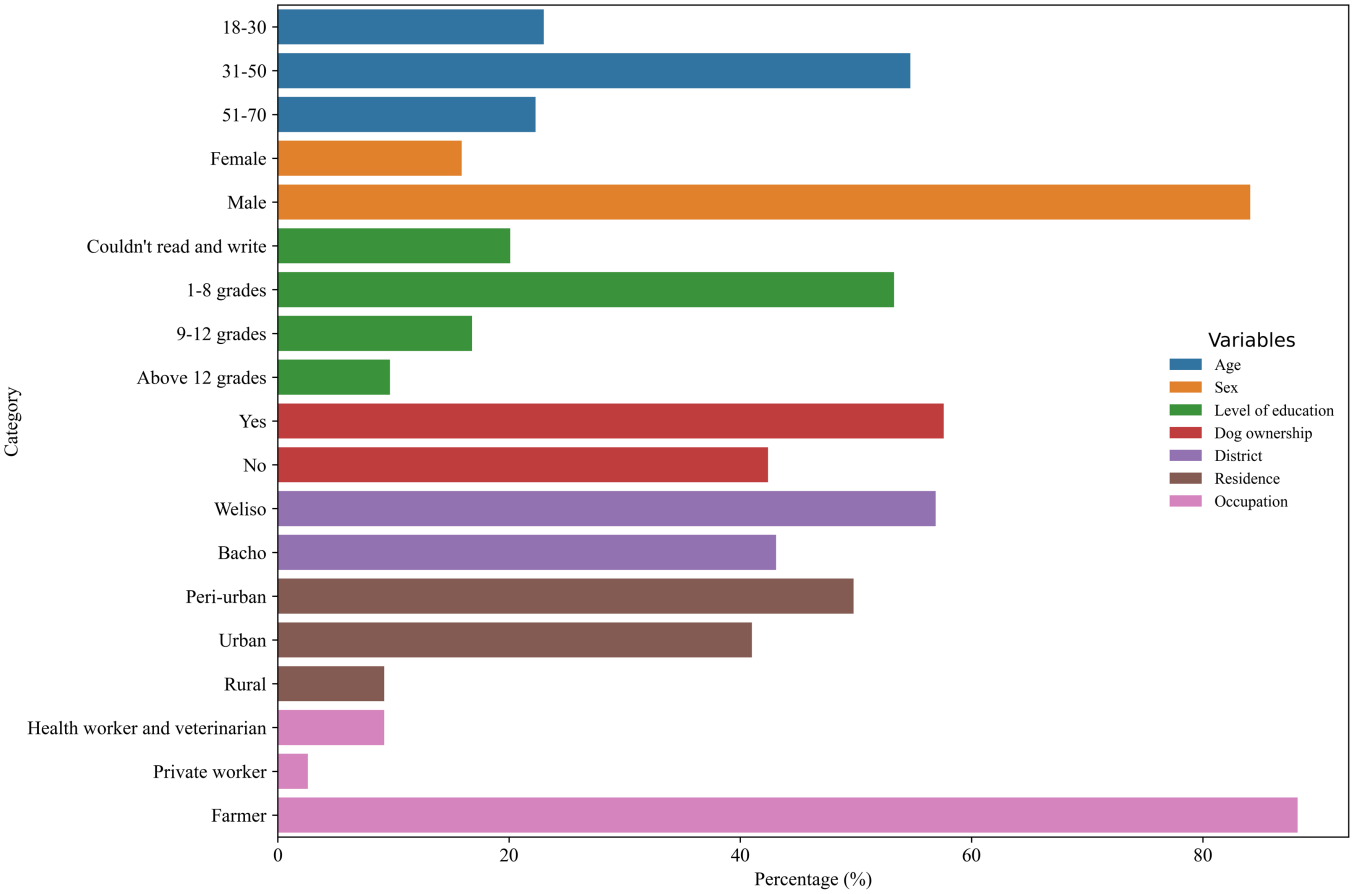
Socio-demographic profiles of the respondents in the study area.

Of 422 study participants, 240 (56.9%) were from the Weliso District, and the remaining 182 (43.1%) were from the Bacho District. Of the total study participants, 355 (84.1%) were men and the rest were women. Most of the participants, 210 (49.8%), lived in the peri-urban area (Figure 3).

### 3.2 Knowledge, attitude, and practice score of the community on rabies

The majority of the respondents, 375 (88.9%), had heard of the disease called rabies, which is locally called “Dhukkuba Saree Maraatuu.” Approximately 287 respondents (68%) reported that dogs are the most affected animals by rabies, followed by bovines [82 (19.4%)], as indicated in Supplementary Table S1.

The study revealed that more than half of the respondents, 246 (58.3%), had good knowledge about rabies. The association between independent variables and knowledge score on rabies was analyzed using logistic regression. According to multivariable logistic regression analysis, informants having a dog (OR = 1.7; 95% CI: 1.050–2.873), living in the urban area (OR = 10.7; 95 CI: 1.106–103), and having an educational level above grade 12 (OR = 2.4; 95% CI: 1.061–5.513) were found to be statistically significantly associated with knowledge score of rabies as shown in Table 1.

**Table 1:** Univariable and multivariable logistic regression analyses between knowledge score on rabies and risk factors in the Southwest Shewa zone.

Variables	Knowledge level		Univariable analysis		Multivariable analysis	
	Poor	Good	OR (95% CI)	*P*-value	OR (95% CI)	*P*-value
**Dog ownership**						
No	105	74				
Yes	71	172	3.4 (2.290–5.159)	0.000	1.7 (1.050–2.873)	0.032
**District**						
Bacho	75	107				
Weliso	101	139	1 (0.653–1.426)	0.857		
**Residence**						
Peri-urban	52	158				
Urban	87	86	0.3 (0.211–0.501)	0.000	10.7 (1.106–103)	0.042
Rural	37	2	0.7 (0.004–0.076)	0.000	2.8 (0.289–28.9)	0.366
**Age**						
18–30	49	48				
31–50	92	139	1.5 (0.957–2.486)	0.075	0.5 (0.242–1.099)	0.086
51–70	35	59	1.7 (0.966–3.065)	0.065	0.9 (0.514–1.644)	0.777
**Sex**						
Female	40	27				
Male	136	219	2.3 (1.400–4.065)	0.001	1 (0.549–2.171)	0.802
**Education level**						
Illiterate	37	48				
Primary	76	149	1.5 (0.907–2.512)	0.313		
Secondary	26	45	1.3 (0.699, 2.545)	0.382		
Above 12	37	4	0.06 (0.027–0.255)	0.000	2.4 (1.061–5.513)	0.036
**Occupation**						
Government employee	37	2				
Private worker	4	7	32.4 (4.941–212)	0.000	0.19 (0.021–1.785)	0.147
Farmer	135	237	32.5 (7.707–136.9)	0.000	1.1 (0.218–5.247)	0.933
**Source of information**						
Family member	107	212				
Mass media	9	15	0.84 (0.357–1.985)	0.693		
Mixed source	60	19	0.16 (0.091–0.281)	0.000	0.4 (0.175–0.788)	0.010
**Previous exposure**						
Yes	143	175				
No	33	71	1.7 (1.101–2.808)	0.018	1.3 (0.752–2.365)	0.325

As depicted in Supplementary Table S2, the results indicated that most of the participants, 371 (87.9%), identified rabies as a health risk, and only 193 (45.7%) knew that prevention of rabies could be achieved through vaccination. More than 70% of the participants believed in the curative nature of traditional medicine.

The majority, 316 (74.9%), of the study participants’ preference in handling the case was taking their diseased animal to the veterinary clinic. Of all respondents, only 202 (47.9%) had positive attitudes toward rabies, whereas the remaining had negative attitudes. The level of positive attitude was significantly higher in private workers (OR = 29.5; 95% CI: 1.809–2.482) than in government employees on multivariable analysis, as illustrated in Table 2.

**Table 2:** Univariable and multivariable logistic regression analyses between attitude score on rabies and risk factors in Southwest Shewa zone.

Variables	Level of attitude		Univariable analysis		Multivariable analysis	
	Negative	Positive	OR (95% CI)	*P*-value	OR (95% CI)	*P*-value
**Dog ownership**						
No	99	80				
Yes	121	122	1.2 (0.847–1.838)	0.263		
**District**						
Bacho	101	81				
Weliso	119	121	1.3 (0.861–1.866)	0.259		
**Residence**						
Peri-urban	121	89				
Urban	64	109	2.3 (1.533–3.498)	0.000	1.8 (1.021–3.215)	0.042
Rural	35	4	0.2 (0.053–0.453)	0.001	5.6 (0.478–66.18)	0.170
**Age**						
18–30	59	38				
31–50	122	109	1.4 (0.856–2.248)	0.018	1 (0.502–1.597)	0.708
51–70	39	55	2.2 (1.228–3.904)	0.008	1.2 (0.574–2.407)	0.659
**Sex**						
Female	43	24				
Male	177	178	1.8 (1.049–3.095)	0.033	1.3 (0.691–2.622)	0.382
**Education level**						
Illiterate	43	42				
Primary	105	120	1.2 (0.710–1.928)	0.538		
Secondary	35	36	1.1 (0.561–1.978)	0.872		
Above 12	37	4	0.1 (0.036–0.338)	0.000	0.3 (0.046–2.540)	0.286
**Occupation**						
Government employee	37	2				
Private work	4	7	49.7 (2.982–829.12)	0.007	29.5 (1.809, 2.482)	0.018
Farmer	179	193	26.8 (2.126–338.9)	0.011	13.6 (0.971–191.2)	0.053
**Source of information**						
Family member	149	170				
Mass media	10	14	1.2 (0.529–2.845)	0.633		
Mixed source	61	18	0.3 (0.146–0.457)	0.000	0.4 (0.203–0.907)	0.003
**Previous exposure**						
Yes	160	158				
No	60	44	0.74(0.475–1.161)	0.392		

The majority, 316 (74.9%), of the study participants’ preference in handling the case was taking their diseased animal to the veterinary clinic. The community’s practice indicator variables toward rabies are depicted in Supplementary Table S3.

In this study, approximately 229 (54.3%) had good prevention practices toward rabies, whereas the remaining 193 (45.7%) of the respondents had poor practices regarding rabies. The practice level was significantly higher in individuals found in the age category of 31–50 years (OR = 1.8; 95% CI: 1.162–3.045) and 51–70 years (OR = 2.7; 95% CI: 1.529–4.942) and male participants (OR = 1.8; 95% CI: 1.071–3.085), as presented in Table 3.

**Table 3:** Univariable and multivariable logistic regression between practice score on rabies and risk factors in Southwest Shewa zone.

Variables	Practice level		Univariable analysis		Multivariable analysis	
	Poor	Good	OR (95% CI)	*P*-value	OR (95% CI)	*P*-value
**Dog ownership**						
No	83	96				
Yes	110	133	1 (0.701–1.540)	0.822		
**District**						
Bacho	113	69				
Weliso	80	160	3.2 (2.191–4.897)	0.000	10 (4.099–24.56)	0.000
**Residence**						
Peri-urban	93	117				
Urban	65	108	2.7 (1.492–5.159)	0.001	2.9 (1.596–5.407)	0.000
Rural	35	4	0.9 (1.102–9.310)	0.000	1 (0.076–7.355)	0.802
**Age**						
18–30	58	39				
31–50	102	129	1.8 (1.162–3.045)	0.010	1 (0.417–1.733)	0.655
51–70	33	61	2.7 (1.529–4.942)	0.001	1 (0.481–2.607)	0.792
**Sex**						
Female	39	28				
Male	154	201	1.8 (1.071–3.085)	0.027	1 (0.609–2.417)	0.582
**Education level**						
Illiterate	42	43				
Primary	87	138	1.5 (0.937–2.562)	0.088	1.5 (0.829–317)	0.178
Secondary	27	44	1.6 (0.839–3.021)	0.155	1 (0.504–2.502)	0.777
Above 12	37	4	0.1 (0.035–0.322)	0.000	0.11 (0.009–0.265)	0.038
**Occupation**						
Government employee	36	3				
Private work	1	10	120 (11.23–128.6)	0.000	16.7 (0.884–317.6)	0.060
Farmer	156	216	16.6 (5.026–54.93)	0.000	1.3 (0.112–15.98)	0.820
**Source of information**						
Family member	126	193				
Mass media	15	9	0.4 (0.166–0.992)	0.032	1 (0.181–1.162)	0.100
Mixed sources	52	27	0.3 (0.202–0.568)	0.000	1.3 (0.593–2.819)	0.518
**Previous exposure**						
Yes	166	152				
No	27	77	3.1 (1.970–5.086)	0.000	1.7 (0.986–3.194)	0.056

### 3.3 Retrospective study of rabies in humans and animals

A total of 5,964 human rabies-suspected cases were recorded from health centers of the zone during the study period. From this, a total of 529 rabies-suspected patients were recorded specifically at Weliso and Bacho Hospitals and health centers from January 2017 to January 2021 on the case book. Rabies occurred with an average of 106 cases registered annually in the health centers of the districts. Most of the cases were recorded in 2019 (160, 30.2%), and most of them were bitten by animals of unknown ownership (328, 62%). The retrospective data on rabies in the study districts are indicated in Table 4.

**Table 4:** Retrospective data on rabies-suspected individuals in both districts.

Variables	Number of cases	Percentage (%)
**Age**		
Children (2–15)	222	42
Young (16–29)	140	26.5
Adult (30–43)	89	16.8
Elder (44–70)	78	14.7
**Sex**		
Male	328	62
Female	201	38
**Residence**		
Peri-urban	179	33.8
Rural	133	25.1
Urban	217	41
**Source of bite**		
Dog	503	95.1
Fox	7	1.3
Cat	13	2.5
Donkey	2	0.4
Horse	4	0.8
**Owner of the animal**		
Known	201	38
Unknown	328	62
**Site of bite**		
Body	39	7.4
Hand	110	20.8
Head	7	1.3
Legs	336	63.5
Multiple sites	6	5.9
Others	31	5.9

This study recorded more rabies-suspected cases in the Weliso district than in Bacho district. The total number of rabies-suspected individuals who took PEP in both districts was almost similar, except in 2019, when the maximum number of cases was recorded, as presented in Supplementary Figure S1.

Compared to other seasons, the highest number of human suspected rabies cases occurred in autumn and winter, as shown in Supplementary Figure S2.

The occurrence of rabies was higher in humans than in animals in the study area. A total of 127 suspected rabies cases were registered in animals. The highest number of suspected rabies cases, 85 (66.9%), was found in bovines, followed by equines and canines. The occurrence of rabies in the various animal species in the districts is summarized in Table 5.

**Table 5:** Rabies reported in animals in the study area.

Year	Number of cases		Suspected animal species for rabies		
	Cases	Percentage (%)	Bovine	Equine	Canine
2017	9	7.1	8	1	0
2018	26	20.5	14	7	5
2019	30	23.6	18	7	5
2020	27	21.3	20	5	2
2021	35	27.6	25	4	6
Total	127	100	85	24	18

### 3.4 Epidemiological distribution and incidence proportion of rabies in humans and animals

The incidence of rabies in humans was higher in 2019 (66.9 cases per 100,000 population) and lower in 2018 (30.5 cases per 100,000 population). The incidence of rabies in humans and animals is presented in Tables 6, 7, respectively. Moreover, approximately nine death cases were recorded in the human population in 5 years (2017–2021), and the death rate calculated was 1.9 cases per 100,000 population.

**Table 6:** Incidence of suspected human rabies in the study area (2017–2021).

Districts	Periods	Rabies cases	Total human population	Incidence/100,000 in humans
Weliso and Bacho	2017	109	213,618	51 cases
Weliso and Bacho	2018	69	226,394	30.5 cases
Weliso and Bacho	2019	160	239,242	66.9 cases
Weliso and Bacho	2020	90	246,631	36.5 cases
Weliso and Bacho	2021	101	254,939	39.6 cases
Weliso and Bacho	2017–2021	529	1,180,824	44.9 cases

**Table 7:** Incidence of suspected rabies in animals in the study area (2017–2021).

District	Periods	Rabies cases	Total animal population	Incidence/100,000 in animals
Weliso and Bacho	2017	9	653,421	1.4 cases
Weliso and Bacho	2018	26	698,536	3.7 cases
Weliso and Bacho	2019	30	723,491	4.1 cases
Weliso and Bacho	2020	27	829,803	3.3 cases
Weliso and Bacho	2021	35	843,782	4.1 cases
Weliso and Bacho	2017–2021	127	3,749,033	3.4

**Figure 4:**
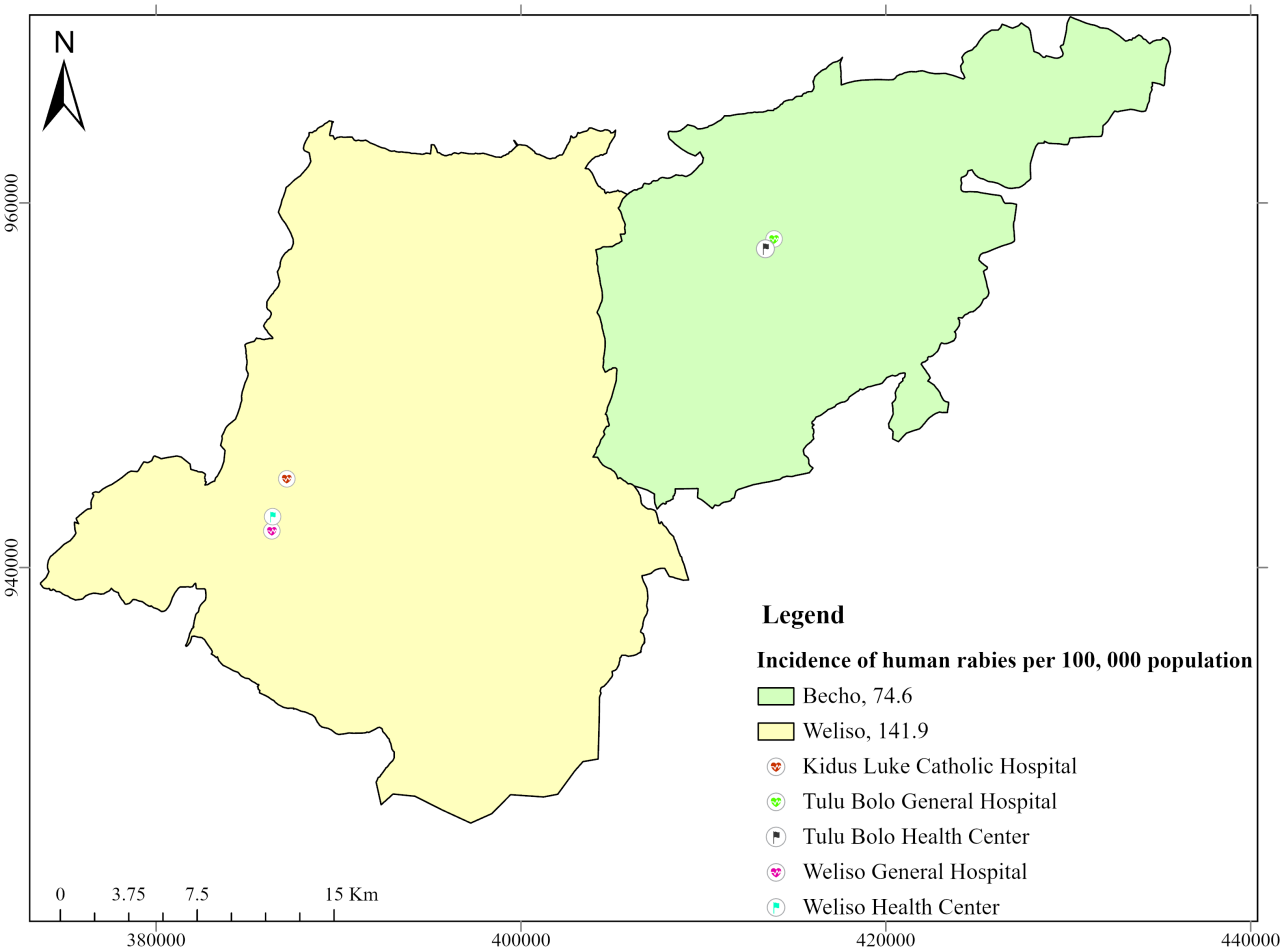
Map showing the incidence of rabies in humans (2017–2021) and respective health infrastructures in the study districts.

The spatial distribution of rabies was indicated using ArcGIS software’s functionalities; the results are presented in Figure 4. The incidence of rabies in humans was higher in the Weliso district (141.9 cases per 100,000 populations) than in the Bacho district (74.6 cases per 100,000 populations).

Rabies incidence cases were higher during the autumn, with 40.9 cases per 100,000 population seasons in both study districts. The highest number of rabies-suspected cases, 68.5 cases per 100,000 populations, was reported in male individuals than in female individuals.

**Figure 5:**
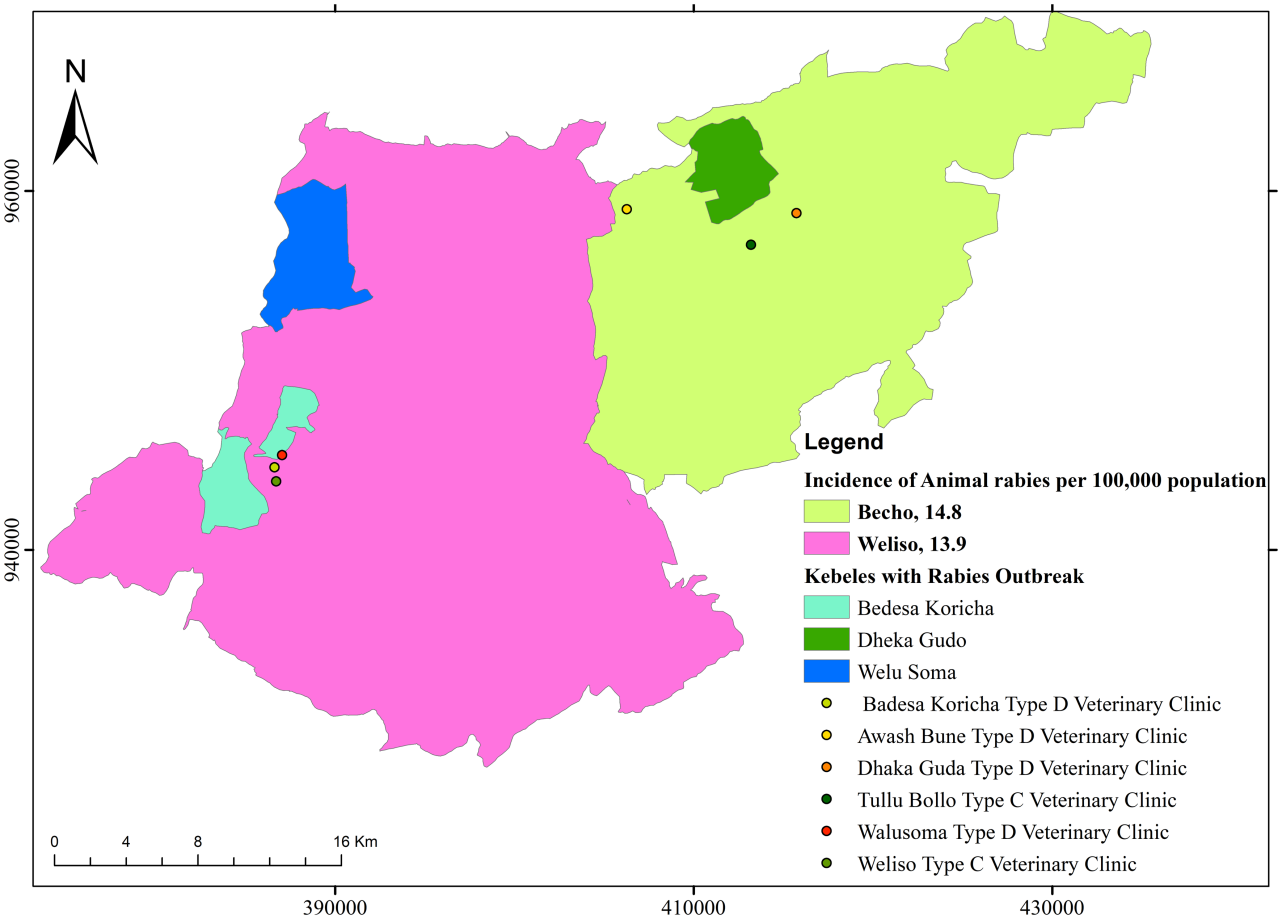
Map showing the incidence of suspected rabies in animals (2017–2021) and respective veterinary infrastructures in the study districts.

The incidence of rabies in animals was higher in the Bacho district (14.8 cases per 100,000 populations) than in the Weliso district (13.9 cases per 100,000 populations), as depicted in Figure 5. The highest incidence was recorded in 2021 (4.1 cases/annually per 100,000 population), whereas the lowest incidence was recorded in 2017 in both study districts (1.4 cases/annually per 100,000 population).

## 4 Discussion

Nowadays, rabies remains a major public and veterinary health risk in most developing countries, including Ethiopia. Designing effective rabies prevention and control programs requires an understanding of levels of knowledge, attitudes, and practices regarding the disease within local communities. Accordingly, this study aimed to assess the knowledge, attitude, and practice and to estimate the incidence and spatial distribution of rabies in humans and animals in the Southwest Shewa zone of Oromia, Ethiopia. The result revealed that in the study area, the level of good knowledge, positive attitude, and good prevention practices was found to be 58.3, 47.9, and 54.2%, respectively.

The level of knowledge about rabies in the study area was similar to the findings of studies conducted in Ethiopia (15, 22, 28). However, this finding is higher than studies conducted in the Massingir District of Mozambique and Thailand (9, 16); on the other hand, the result was lower than studies conducted in different parts of Ethiopia such as Tigray Region, Bahir Dar city, and Debre Tabor town (25, 29, 30). The difference could be because of the socio-demographic characteristics such as educational status, information access of the community, and tool differences. The other possible reasons for the difference could be due to low health promotion, particularly regarding rabies in this study area.

This study pointed out that the level of positive attitude was 47.9%. This figure is similar to studies in Ambo town (22) and Debre Tabor town (29) of Ethiopia. However, the findings are lower than studies in Thailand and different parts of Northern Ethiopia (15, 16, 25, 28, 30). Conversely, the finding is higher than that of studies conducted in Mozambique (9), and the difference could be because of study time, sample size, tool, and differences in socio-demographic characteristics. This could also be due to sample size differences and access to different information about the disease in the area.

Concerning the level of good prevention practices toward rabies, it was found to be 54.2% in the current study. This finding is similar to studies in Thailand and different regions of Ethiopia (16, 22, 29), but the current finding is higher than the good practice level in studies in the Massingir district of Mozambique (9). Contrary to this, the current figure is lower than findings in studies conducted in different regions of Ethiopia (15, 25, 28, 30); the difference could be because the current included districts with rural communities, unlike those studies. This could be because of a low level of knowledge, which might result in a low level of good practice. Lack of access, lack of awareness on vaccination, absence of veterinary clinics in some areas, and cost and availability of vaccines could also be the possible reasons.

Household heads with a dog were 1.7 times more likely to have a good knowledge score than non-dog owners. The statistically significant difference (*p* = 0.032) in knowledge score between dog owners and non-dog owners could be attributed to the fact that dog owners have a better chance to know more about dogs and dog diseases. This finding was also supported by many scholars who mentioned awareness level as a useful tool to control rabies (15, 31). Respondents who live in urban areas were 10.7 and 1.8 times more likely to have higher good knowledge and cheerful outlook scores than peri-urban residents, respectively. The higher knowledge score in urban residents was in line with the previous findings in the Amhara region, Ethiopia, by Bahiru et al. (28). Informants above grade 12 with good knowledge scores were 2.4 times more likely to have better knowledge than illiterate. This finding was also supported by a study conducted in the country (29, 30) and elsewhere (32). This could be because educated people have better access to information and can easily understand the disease.

In this study, 529 suspected human rabies cases were documented between 2017 and 2021. This finding is significantly lower compared to the studies carried out in Jimma zone (33), Northwestern Tigray (34), and Addis Ababa (35), which reported 2,180, 2,302, and 1,772 human rabies exposure cases by dog bites, respectively. Increasing public awareness, improved health-seeking behavior, and improved reporting systems may have contributed to the increase in reported cases of human rabies caused by dog bites.

More suspected rabies cases were reported in humans (529 cases) than in animals (127 cases). This might be associated with a lack of awareness and a weak disease-reporting system in the veterinary sector. This finding disagrees with study conducted by Reta (36), who registered a higher number of rabies cases (2,337 cases) in animals in and around Addis Ababa. Human rabies death cases (1.9 cases per 100,000 population) reported were relatively lower than those in a study by Mengistu et al. (19), which found 9.3 cases per 1,000 population in the Tigray region between 2009 and 2012. This could be due to the high dog population, the poor trend of the communities to vaccinate their dogs, the sample size, and the lack of awareness about the disease.

The highest incidence of rabies cases found in humans occurred in the Weliso district (44.99 cases per 100,000 people). This could be due to the high dog population, poor trend of the communities to vaccinate their dogs, and lack of awareness of prevention ways of the disease. The study indicated that individuals within the age groups of 2 and 15 were more affected by rabies. This finding was corroborated by data from the World Health Organization, which showed that most (30–50%) of the victims of rabies reported from Africa and Asia were children (37).

Most of the rabies cases occurred during the autumn season, which is associated with a suitable breeding season for dogs in Ethiopia, which favors the transmission of the disease. The finding agrees with the study done by Deressa et al. (7) in Ethiopia. In addition, the study conducted by Mengistu et al. (38) highlighted that rabies transmission is greatly aided by a combination of large dog population during the breeding season, low vaccination rates, and a lack of public awareness, particularly in rural areas.

Unknown-owner rabid dogs were found to be responsible for the bites of 503 (95.1%) suspected rabies cases. This is consistent with the study conducted in Jimma Town (39), which indicated that a considerable proportion of the interviewed households (97.2%) suggested rabies is transmitted to humans when they are bitten, scratched, or licked by rabid dogs. Another study conducted by Kitala et al. (40) also reported that 97% of humans used post-exposure treatments to rabies because of dog bite in Kenya. The occurrence of rabies did not follow any trend between these study periods (no increasing or decreasing trend). This could be due to the lack of continuous health promotion services and measures undertaken by the government to control the disease. The majority of rabies-suspected individuals, 365 (69%), received post-exposure prophylaxis (nerve tissue vaccine). The finding is in line with the study done by Mesfin (35) in Addis Ababa and surrounding towns in which most of the patients took nerve tissue vaccine.

## 5 Conclusion

In conclusion, the current study revealed that the level of knowledge, positive attitude, and good preventive practice toward rabies was inadequate. In this study, the level of good practice was found to be slightly higher than attitude. One can expect that, after a positive attitude, good practice will come. Rabies knowledge scores were influenced by dog ownership, residence, and education level. The site of living and occupation are two risk factors linked to an attitude score. Even though the assessment of the disease was derived from limited, insufficient, and sparse data, the present mapping demonstrated the incidence and extent of diseases in the research region. It also indicated that no efforts have been made to combat the disease’s spread and transmission. To control and raise community awareness in the study area, intersectoral collaboration—particularly the implementation of a One Health approach between the public health and veterinary sectors—is crucial. Public education and awareness campaigns, mass dog vaccination, improved access to PEP, and routine vaccination are also essential. To determine the true picture of the disease, enhanced surveillance and rigorous research on current data are also required, and the possibility of using a computer-based case recording database system has been entertained.

## Data Availability

The raw data supporting the conclusions of this article will be made available by the authors, without undue reservation.

## Supplementary material

SUPPLEMENTARY FIGURE S1 Temporal distribution of human suspected rabies in the study area.

SUPPLEMENTARY FIGURE S2 Seasonal distribution of suspected rabies in humans.
