# Supplementary figures and images for: Community knowledge, attitude, and practice, incidence of suspected cases, and epidemiological distribution of rabies in humans and animals in Southwest Shewa zone, Oromia, Ethiopia
Source: Front Vet Sci. 2025 Apr 8;12:1448448. doi: 10.3389/fvets.2025.1448448 (PMC12013722; doi:10.3389/fvets.2025.1448448)

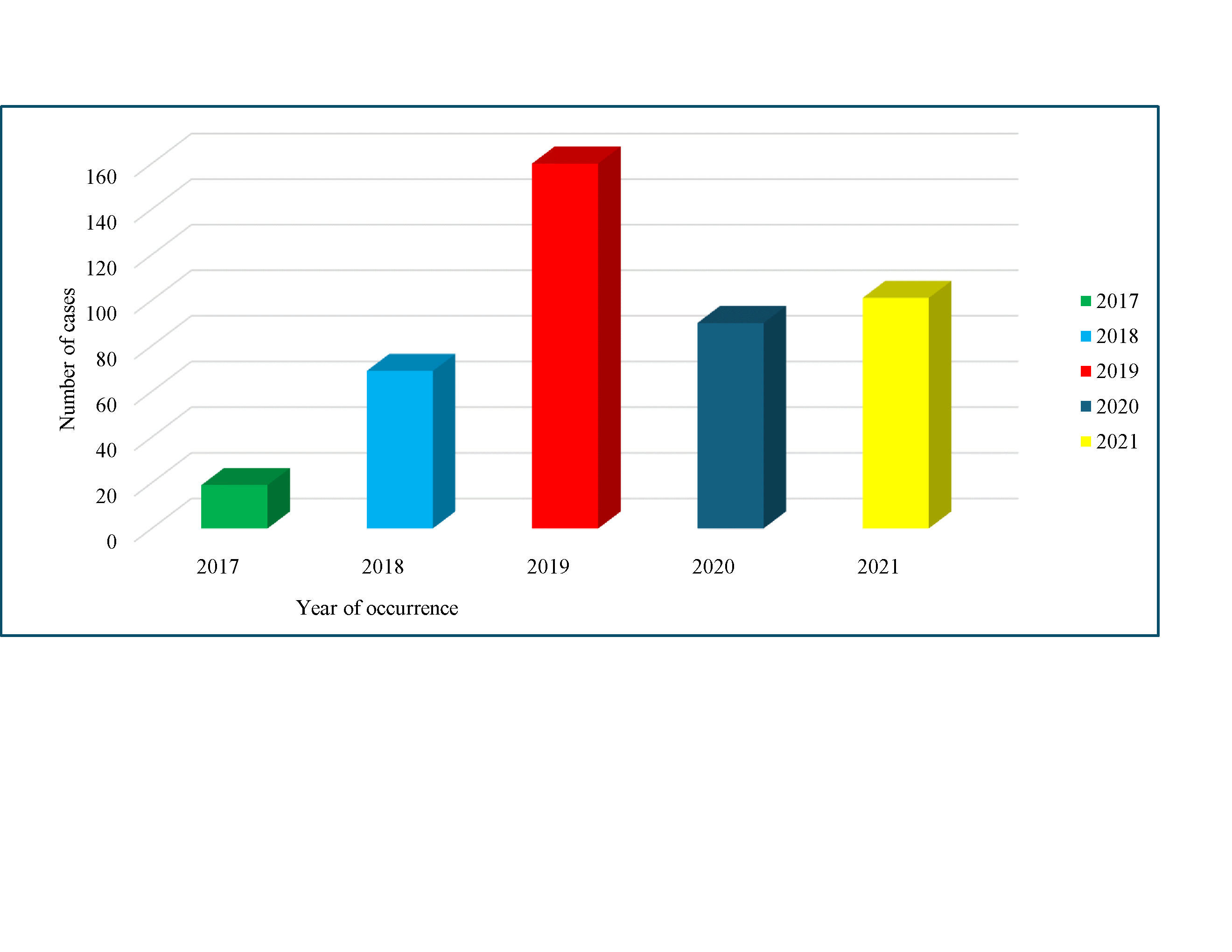

Supplement: SUPPLEMENTARY FIGURE S1 — Temporal distribution of human suspected rabies in the study area. [file Image_1.TIF]
