# Supplementary material for: Community knowledge, attitude, and practice, incidence of suspected cases, and epidemiological distribution of rabies in humans and animals in Southwest Shewa zone, Oromia, Ethiopia
Source: Front Vet Sci. 2025 Apr 8;12:1448448. doi: 10.3389/fvets.2025.1448448 (PMC12013722; doi:10.3389/fvets.2025.1448448)
Supplement: Supplementary file 3 [file Table_1.docx]

**Supplementary Table 1** - **Community’s knowledge indicator variables on rabies in Southwest Shewa Zone**

| **Knowledge** | **Response category** | **Total (%)** |
| --- | --- | --- |
| Ever heard of rabies | Yes | 375/88.9 |
|  | No | 47/11.3 |
| Awareness about rabies | Rabies described as a disease | 337/79.9 |
|  | Disease that affects the CNS of animals and humans | 59/20.1 |
|  | Unknown/wrong answer | 26/6.2 |
| Rabies is a fatal disease | Yes | 286/67.8 |
|  | No | 122/28.9 |
|  | Not sure | 14/3.3 |
| Cause of rabies | Germ | 234/55.4 |
|  | Evil eye | 28/6.6 |
|  | Satan | 16/3.8 |
|  | Don’t know | 144/34.2 |
| Transmit from animal to human | Yes | 336/79.6 |
|  | No | 72/17.1 |
|  | Not sure | 14/3.3 |
| Mode of transmission | Rabid dog/cat bite | 225/53.3 |
|  | Through scratch | 111/26.3 |
|  | Unknown/wrong answer | 86/20.34 |
| Common outbreak season | Summer | 94/22.3 |
|  | Autumn | 143/33.9 |
|  | Spring | 59/13.9 |
|  | Winter | 126/29.9 |
| Source of information | Family member | 319/75.6 |
|  | Mass media | 24/5.7 |
|  | Mixed source | 79/18.7 |
| Symptoms of rabies | Behavioral change | 203/48 |
|  | Excessive salivation | 37/8.8 |
|  | Barking like dog | 37/8.8 |
|  | Don’t know | 145/34.3 |
| Preventable disease | Yes | 266/63 |
|  | No | 156/37 |
| Common reservoir/s | Dog | 60/14.2 |
|  | Fox | 224/53 |
|  | Don’t know | 103/24.4 |
|  | Night bat | 3/0.7 |
|  | Wild animals | 32/7.6 |
