# Supplementary material for: Community knowledge, attitude, and practice, incidence of suspected cases, and epidemiological distribution of rabies in humans and animals in Southwest Shewa zone, Oromia, Ethiopia
Source: Front Vet Sci. 2025 Apr 8;12:1448448. doi: 10.3389/fvets.2025.1448448 (PMC12013722; doi:10.3389/fvets.2025.1448448)
Supplement: Supplementary file 4 [file Table_2.docx]

Supplementary Table 2 - Community’s attitude indicating variables on rabies in Southwest Shewa Zone

| **Variables** | **Category** | **Response** | **Percent (%)** |
| --- | --- | --- | --- |
| Rabies is a health risk | Yes | 371 | 87.9 |
|  | No | 51 | 12.1 |
| Do you seek medical evaluation immediately after bitten | Yes | 364 | 86.3 |
|  | No | 58 | 13.7 |
| Willingness to vaccinate | Yes | 211 | 86.8 |
|  | No | 32 | 13.2 |
| Ways of rabies prevention | Using holly water | 42 | 10 |
|  | Using traditional medicine | 38 | 9 |
|  | Using vaccination | 342 | 81 |
| Traditional medicine can cure rabies | Yes | 298 | 70.6 |
|  | No | 124 | 29.4 |
| Preferable treatment | Modern drugs  Traditional drugs | 170  252 | 40.3  59.7 |
| Rabies is zoonotic | Yes | 339 | 80.3 |
|  | No | 83 | 19.7 |
