# Supplementary material for: Community knowledge, attitude, and practice, incidence of suspected cases, and epidemiological distribution of rabies in humans and animals in Southwest Shewa zone, Oromia, Ethiopia
Source: Front Vet Sci. 2025 Apr 8;12:1448448. doi: 10.3389/fvets.2025.1448448 (PMC12013722; doi:10.3389/fvets.2025.1448448)
Supplement: Supplementary file 5 [file Table_3.docx]

Supplementary Table 3 - Community’s practice indicator variables on rabies

| **Variables** | **Category** | **Frequency (%)** |
| --- | --- | --- |
| Do you have a dog | Yes | 243/57.4 |
|  | No | 179/42.4 |
| Purpose of keeping dogs | As guard of house | 226/53.5 |
|  | For hunting | 5/1.2 |
|  | For crop protection | 12/2.8 |
|  | Non dog owner | 179/42.42 |
| Dog management practice | Free in the field | 96/22.75 |
|  | Kept indoor | 54/12.8 |
|  | Housed in cages | 34/8.05 |
|  | Tie outside | 59/14 |
|  | Non dog owner | 179/42.4 |
| Handling food animals diseased from rabies | Take to vet clinic | 316 (74.9) |
|  | Give traditional drugs | 9 (2.1%) |
|  | Slaughter, cook and eat | 50 (11.8) |
|  | None | 47 (11.1) |
| Dead animal management | Burn | 235 (55.7) |
|  | Bury | 121 (28.7) |
|  | Leave open | 7 (1.7) |
|  | Eat | 12 (2.8) |
|  | None | 47 (11.1) |
| Outbreak management | Avoid contact | 227 (53.8) |
|  | Isolation | 113 (26.8) |
|  | Vaccination | 33 (7.8) |
|  | Neither | 49 (11.6) |
| Do you have vaccinated your dogs? | Yes | 48/11.4 |
|  | No | 195/46.2 |
|  | Non dog owner | 179/42.4 |
| Importance of vaccine | Yes | 213/50.5 |
|  | No | 30/7 |
|  | Not sure | 179/42.4 |
| Immediate measure against a symptomatic case | Kill and destroy | 212 (50.2) |
|  | Take/report to clinic | 163 (38.6) |
|  | None | 47 (11.1) |
